# Lipid Peroxidation-Related Redox Signaling in Osteosarcoma

**DOI:** 10.3390/ijms25084559

**Published:** 2024-04-22

**Authors:** Suzana Borović Šunjić, Morana Jaganjac, Josipa Vlainić, Mirna Halasz, Neven Žarković

**Affiliations:** Laboratory for Oxidative Stress, Division of Molecular Medicine, Ruder Boskovic Institute, Bijenicka 54, 10000 Zagreb, Croatia; morana.jaganjac@irb.hr (M.J.); josipa.vlainic@irb.hr (J.V.); mirna.halasz@irb.hr (M.H.)

**Keywords:** lipid peroxidation, reactive aldehydes, redox signaling, osteosarcoma

## Abstract

Oxidative stress and lipid peroxidation play important roles in numerous physiological and pathological processes, while the bioactive products of lipid peroxidation, lipid hydroperoxides and reactive aldehydes, act as important mediators of redox signaling in normal and malignant cells. Many types of cancer, including osteosarcoma, express altered redox signaling pathways. Such redox signaling pathways protect cancer cells from the cytotoxic effects of oxidative stress, thus supporting malignant transformation, and eventually from cytotoxic anticancer therapies associated with oxidative stress. In this review, we aim to explore the status of lipid peroxidation in osteosarcoma and highlight the involvement of lipid peroxidation products in redox signaling pathways, including the involvement of lipid peroxidation in osteosarcoma therapies.

## 1. Introduction

Osteosarcoma (OS) is the most common primary malignant bone tumor of mesenchymal origin and is relatively rare in the general population. It is common in two age groups: in children from early childhood to young adults, and later in adults over the age of 50. OS most commonly occurs in the long bones, but other bones can also be affected [1,2]. The median survival time of patients with OS is 39 months, and the 5-year survival rate is 58.0% [2]. In children and adolescents, the 5-year survival rate is between 64 and 78%, depending on the type of OS and age group [3].

Some inherited genetic mutations are associated with an increased risk of developing OS, e.g., mutations of genes such as tumor suppressor *p53* (*P53*) [4]; retinoblastoma protein (RB); ATP-dependent DNA helicase Q4 (RECQL4); Bloom syndrome, RecQ Helicase-Like (BLM); and Werner syndrome, RecQ Helicase-Like (WRN) [5,6,7,8]. OS shows a very heterogenous appearance and no single gene mutation is responsible for the development of OS [9,10]. It appears that this broad spectrum of genetic alterations, including gains, losses, or rearrangements of chromosomal regions, is related to disrupted osteoblastic differentiation [7]. Altered gene expression and changes in signaling pathways may contribute to the malignant transformation of OS [11]. OS, as well as other carcinomas, manifest different metabolic phenotypes to sustain growth and survival. Metabolic profile as well as lipid peroxidation can be impacted, among others, by epigenetic alterations [12,13,14].

Current OS therapies are based on a basic approach based on chemotherapy, usually doxorubicin, methotrexate, and cisplatin in combination before or after surgery. Radiotherapy is also being considered, although OS is quite resistant, while other approaches (such as adjuvant immunotherapy), although with some good results, are still under trial [15,16]. Treatment of other cancers with radiotherapy can, as a consequence, cause the development of OS in neighboring tissues [17].

The anticancer effect of chemotherapy and radiotherapy is often based on the induction of oxidative stress [18] and the formation of reactive oxygen species (ROS), which subsequently damage the cancer cells [19,20]. However, redox homeostasis in cancer cells keeps ROS higher than in normal cells, and cancer cells use this excess of ROS to promote proliferation, migration, and metastasis [21]. This excess of ROS makes cancer cells more susceptible to the additional ROS created by chemotherapy and radiotherapy. However, an adaptive antioxidant system could combat oxidative stress, leading to resistance to chemotherapy and radiotherapy [22,23].

Although excessive levels of ROS lead to cell death, sublethal levels of ROS will not have detrimental effects on cells but instead, the cells will survive with affected cellular proteins, lipids, and DNA. Various redox signaling cascades associated with DNA damage are then activated, leading to the activation of oncogenes (*H-RAS*) or the inactivation of tumor suppressor genes (*P53*), and thus to the development of cancer [21].

Cancer cells share several properties that help them survive and thrive, and each of these properties is associated with a change in signaling. Cancer growth is supported by sustained proliferative signaling and replicative immortality, escape from growth suppressors and resistance to cell death, changes in cell metabolism, increased mutation, the induction of angiogenesis, the promotion of inflammation and avoidance of destruction by the immune system, invasion, and the formation of metastases [24,25]. These properties are explored in [26] with regard to redox signaling. In this review, we aim to examine the current data on the status of lipid peroxidation in OS, including the involvement of lipid peroxidation in OS therapy. The involvement of lipid peroxidation products in redox signaling pathways will be evaluated in relation to the relevant signaling pathways in OS.

## 2. Osteosarcoma Classification

Research on OS is difficult since it has a complex genome, rather low incidence, and there are significant biologic differences between the subtypes [27]. OS is the most common primary malignant tumor of the connective tissue, having a mesodermal origin. In OS, the tumor cells, originating from bone tissue and only rarely soft tissue, produce bone or osteoid. Based on histology findings on predominant cells, OS can be subdivided into osteoblastic, chondroblastic, and fibroblastic types, although these three cell types can be found in a single tumor. In addition, OS often produces various amounts of cartilage matrix or fibrous tissue [28]. Based on the place of origin, OS cancers are divided into two main groups: primary, which can be intramedullary (central) or surface, and secondary tumors. However, although OS predominantly occurs at the metaphysis and arises from medulla, it may also arise from the bone surface, cortex, or even in an extraskeletal site. Based on its anatomical location, OSs are divided into osseous (central, surface, gnathic, multifocal) and soft tissue (intramuscular and other) OS. The World Health Organization classifies OS to six subtypes: low-grade central OS, OS not otherwise specified, parosteal, periosteal, high-grade surface, and secondary OS.

The diagnosis and prognosis of OS are difficult. Gathered information and different recommendations group OS into scalar systems, whereas histology is most important for oncologic stage determination and therapy prescription. Thus, several grading systems are in use, but the most popular is that of a 4-stage grading system based on Broders’ grading adapted for osteosarcoma by the Mayo Clinic group [29]. Namely, in this Broders’ schema, the numeric grade of the tumor varies from one to four and is associated with the percentage of anaplasia in the tumor (from ≤25% up to 100%). In this schema, the most important characteristic is the cytologic atypia of tumor cells, and most of the central OS is assigned a grade of three or four (high grade). OS histologic grade 1 are low-grade central OS and usually with no anaplasia. In the OS staging schema, the stage also depends on metastasis findings. Based on current knowledge, almost all conventional OS are high-grade tumors. On the contrary, almost all surface OS are low-grade tumors, whereas only some gnathic and periosteal OS are in grade 1, rarely grade 2 or 3. These grades are important to determine the necessity for surgery and chemotherapy [30].

Poor prognosis and high lethality in patients with osteosarcoma are highly associated with metastases. The high propensity of cell spreading results in a relatively high likelihood of the appearance of distant metastases, firstly and most commonly in the lungs and secondly in the lymphatic system, although almost any organ can be affected, so the prognosis for patients with OS is always dismal. Namely, OS cells can locate themselves in a second tissue. These cells eventually differentiate and form a suitable microenvironment for the growth of metastatic OS cells, leading to the formation of metastases. These cells usually differentiate and are not identical to the cells of origin due to specific and various molecular changes [31]. Scientific efforts led to novel findings and the discovery of biochemical and molecular markers involved in OS progress and development, which subsequently improved treatment modalities [5]. 

Some studies demonstrated that ROS generation is a modulator of bone cell function, and that the pathophysiology of mineralized tissues that is influenced by oxidative stress have a great influence on chemotherapy and apoptosis in OS, but can also impact carcinogenesis [32].

## 3. Lipid Peroxidation in Osteosarcoma

Redox reprogramming is among the hallmarks of cancer, and plays a crucial role in the initiation, development, and progression of cancer. Its roles in tumorigenesis and the onset of metastasis have been covered in recent reviews [22,33,34]. Below, redox reprograming in cancer in general and in particular in OS is discussed. 

Cancer is characterized by an altered antioxidant defense and a deregulated redox homeostasis, which leads to the excessive formation and accumulation of ROS and consequently to oxidative stress [35]. Increased ROS have been implicated in both tumor progression and tumor regression. Moderate levels of ROS can reverse or slow the development of tumors, while high levels of ROS accelerate carcinogenesis [36,37,38,39,40]. ROS can have varying effects on macromolecules depending on the site of origin, their reactivity, and diffusion distance [41]. Polyunsaturated fatty acids (PUFAs) are particularly vulnerable to ROS-induced damage at the bis-allylic site, which triggers lipid peroxidation [42]. In addition to non-enzymatic oxidation, lipid peroxidation can also occur by enzymatic oxidation via peroxidases such as phospholipase A2, cyclooxygenase, lipoxygenase, and cytochrome p450 [42]. The type of lipid peroxidation end products depends on the type of PUFA oxidized. Enzymatic lipid peroxidation end products of linoleic acid (omega-6 PUFA) are hydroperoxy octadecadienoates (HPODEs), whereas the enzymatic oxidation of arachidonic acid (omega-6 PUFA) will give rise to prostaglandins, thromboxanes, leukotrienes, lipoxins, hydroperoxyeicosatetraenoic acid, epoxyeicosatrienoic acid, and 20-hydroxyeicosatetraenoic acid [43]. Moreover, lipid hydroperoxides (LOOH) are formed in the early stage of ROS-induced lipid peroxidation. Similar to enzymatic lipid peroxidation, the ROS-induced peroxidation of linoleic acid leads to the formation of HPODEs. Peroxidation of arachidonic acid will give rise to F2-isoprostanes and isofurans, while peroxidation of docosahexaenoic acid (omega-3 PUFA) generates neuroprostanes [44]. Reactive aldehydes, including 4-hydroxyalkenals, 2-alkenals, ketoaldehydes, and other similar unsaturated aldehydes, are among the end products of lipid peroxidation. Some of the well-studied aldehydes derived from lipid peroxidation are 4-hydroxynonenal (4-HNE), malondialdehyde (MDA), and acrolein, with 4-HNE being the best known due to its bioactive properties [45,46,47]. Due to its high reactivity, 4-HNE can interact with different macromolecules and influence various signaling processes and cellular functions [48,49,50,51,52]. 4-HNE, at physiological concentrations, acts as a metabolic regulator, while at higher exogenously added concentrations, it causes dose- and time-dependent cytostatic or cytotoxic effects [53]. Calongi and coworkers demonstrated the cytotoxic effect of 4-HNE on the human OS cell line (SaOS2), inducing apoptosis, cytostatic effects, and growth retardation [54]. Our previous work also showed that 4-HNE inhibits cell proliferation and might be one of the important signaling molecules regulating the growth of human bone or at least OS cells [53]. We have shown that 4-HNE can have a different effect on undifferentiated and differentiated human osteosarcoma cells (HOSs). Differentiated HOS cells exhibited lower levels of *c-Myc* and glutathione (GSH), a marked increase in C20:3 fatty acids, decreased metabolism of 4-HNE, and an elevated amount of proteins modified with 4-HNE compared to undifferentiated cells [55]. It is possible that if malignant OS cells differentiate in a manner that alters lipid metabolism, 4-HNE could act as a natural promoter for cell decay [55]. Indeed, the reprogramming of lipid metabolism and the alteration of the lipidome profile are two of the most important metabolic traits of cancer cells [56]. Fatty acids are not only the building blocks of membranes but also have an important signaling function and are vital fuel sources for cellular energy production [57]. The relative amounts of saturated fatty acids compared to unsaturated fatty acids have been found in cancer tissues and malignant OS cells [55,58]. Tumors have a more active lipid metabolism and, as recently discovered, in addition to the classical desaturase pathway, also have an alternative fatty acid desaturation pathway that supports the biosynthesis of unsaturated fatty acids required for tumor growth [59]. Since cancer cells are exposed to higher levels of oxidative stress than normal cells [60], one could suspect the greater sensitivity of cancer cells to lipid peroxidation-induced cell death via ferroptosis, although this does not seem to be the case. 

Patients with primary bone and soft tissue sarcomas exhibit impaired redox homeostasis, as evidenced by the lower levels of antioxidant defenses such as thiols, catalase, and superoxide, as well as elevated protein carbonyl levels and malondialdehyde in biological fluids [32]. However, the presence of lipid peroxidation end products in tumor tissue varies. In some tumor types, the presence of proteins modified with lipid peroxidation derived reactive aldehydes has been found to correlate with malignancy, whereas in other tumor types, the presence of modified proteins in tumor tissues decreases [46,61], suggesting cellular mechanisms that are critical for cells to avoid lipid peroxidation-induced cell death. The cells have developed various detoxification mechanisms that protect them from lipid peroxidation. 

Cellular antioxidant defense mechanisms decrease the level of pro-oxidants, and alterations in major antioxidant systems in tumorigenesis have recently been reviewed [35]. Besides antioxidants, cells also own other mechanisms for the removal of lipid peroxidation products. For example, the major route for 4-HNE detoxification occurs through enzymatic disposal via glutathione-S-transferase (GST), aldehyde dehydrogenase (ALDH), and alcohol dehydrogenase, with GSTA4 having the highest catalytic activity. Conjugation of 4-HNE to GSH via GSTA4 produces glutathionyl-HNE [62] that is eliminated from cells via ATP-dependent RLIP76 export [63,64]. The inability of cells to eliminate lipid peroxidation products would render them prone to ferroptotic cell death. Ferroptosis is a novel form of programmed cell death and is marked by lipid peroxidation and iron overload. This process involves the accumulation of LOOH and is distinct from other forms of cell death, such as apoptosis and necrosis [65]. Ferroptosis is dependent on iron and involves an iron-dependent form of lipid peroxidation. Iron plays a crucial role in the Fenton reaction, where it reacts with hydrogen peroxide to produce highly reactive hydroxyl radicals, initiating lipid peroxidation. In ferroptosis, there is a depletion of GSH, a major cellular antioxidant that helps protect cells from OS. GSH acts as a cofactor for the enzyme glutathione peroxidase 4 (GPX4), which normally protects cells from lipid peroxidation by reducing LOOH to non-toxic lipid alcohols. GPX4 is inhibited in ferroptosis, either due to decreased availability of its cofactor GSH, or due to direct inhibition by specific compounds [66]. When GPX4 is unable to perform its function, lipid peroxidation proceeds unchecked and leads to the accumulation of LOOH in cellular membranes, particularly phospholipids containing PUFAs. This process disrupts the integrity and fluidity of the lipid bilayer, compromising cell structure and function [67,68]. Escape from ferroptosis has recently emerged as a novel hallmark of tumor progression.

Based on the Cancer Genome Atlas sequencing data, a study on the differences in ferroptosis-related gene expression between lung squamous cell carcinoma (LSCC) and adjacent tissue revealed that progression- and disease-free survival might be predicted by the high expression of heat shock protein A5 (HSPA5) [69]. Elevated expression was also found in lung adenocarcinoma patients; however, this did not indicate a better prognosis, while patients with metastatic LSCC who expressed high levels of HSPA5 had shorter overall and progression-free survival times [69]. By preventing ferroptosis, a novel oncogene called tribbles pseudokinase 3 (TRIB3) increases the aggressiveness of head and neck squamous cell carcinoma [70].

Max-like protein X (MLX), a member of the Myc-MLX network, is involved in the lipid metabolism pathway and can cause metabolic reprogramming, and its overexpression is associated with a poor prognosis in OS patients. The MLX knockdown promotes the accumulation of intracellular ferrous iron (Fe^2+^), supporting ferroptosis and affecting OS growth and metastases [71]. Furthermore, in order to facilitate extracellular cysteine (Cys) uptake, which is necessary for GSH biosynthesis, MLX regulates the glutamate/cystine antiporter SLC7A11 [71]. This limits the production of ROS and preserves the redox equilibrium of OS cells. Another study found elevated Fanconi Anemia Complementation Group D2 (FANCD2) in OS cells. The same study demonstrated that FANCD2 knockdown increases the labile iron pool, Fe^2+^, and lipid peroxidation, and inhibits the JAK2/STAT3 pathway, impairing OS cell viability, invasion, and migration, while this is reversed by ferroptosis inhibitor Fer-1 [72]. 

**Figure 1:**
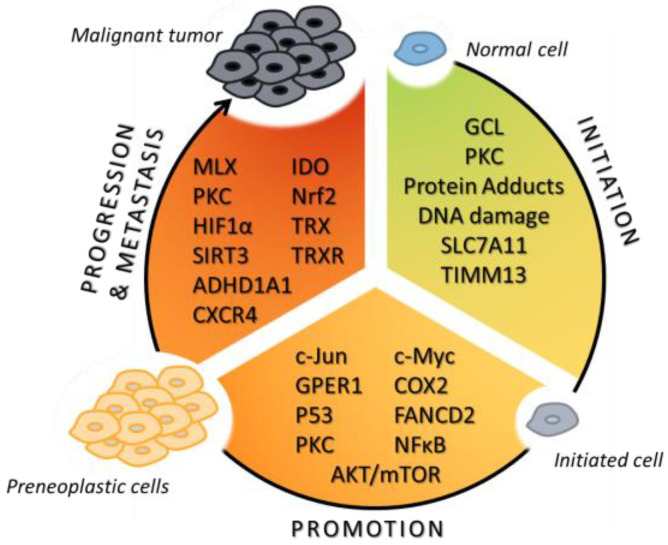
Flow diagram on the involvement of lipid peroxidation in OS tumorigenesis.

Cancer cells mostly obtain ATP through glycolysis rather than mitochondrial oxidative phosphorylation, and mitochondrial dysfunction plays an important role in cancer progression. An inhibitor of mitochondrial transcription 1 (IMT1), which is also an inhibitor of mitochondrial RNA polymerase, was found to promote lipid peroxidation and mitochondrial depolarization by affecting mitochondrial function in OS cells [73]. Moreover, it was found to decrease proliferation and migration in primary and immortalized OS cells and to induce apoptosis in OS cells without causing cytotoxicity in human osteoblasts or osteoblastic cells [73]. This could, at least in part, be attributed to the Akt-mammalian target of rapamycin (mTOR) cascade inhibition by IMT1 [73]. The exposure of human OS cells MG63 to 4-HNE induces caspase-3 activation and modifies the ratio of *Bax/Bcl2* via inactivation of the AKT/mTOR pathway [74]. Similarly, the translocase of inner mitochondrial membrane 13 (TIMM13) is elevated in OS tissues and cells [75]. Its depletion results in oxidative damage, lipid peroxidation, DNA damage, and impaired mitochondrial function, while its overexpression promotes cell motility and proliferation by raising ATP levels. In primary OS cells, TIMM13 depletion inhibits Akt-mTOR activation [75]. The transcription of TIMM13, which is dependent on Homeobox C13, is markedly elevated in OS tissues and cells, highlighting its importance for OS progression [75]. A recent study revealed that G protein-coupled estrogen receptor 1 also prevents ferroptosis in non-small cell lung cancer by activating PI3K/AKT/mTOR signaling, thereby inducing stearoyl CoA desaturase 1 (SCD1) expression [76]. From all the above, and as ferroptosis is regulated by mitochondrial iron metabolism, altered mitochondrial function in cancer seems to be among the mechanisms that protect cells from ferroptosis. A flow chart summarizing the tumorigenesis-associated signaling pathways that may be regulated by lipid peroxidation is shown in Figure 1.

Solid tumors are prone to infiltration by macrophages, which polarize into tumor-associated macrophages (TAMs) to support tumor progression. TAMs increase the expression of ceruloplasmin mRNA, which is transferred to tumor cells via extracellular vesicles. It was shown that this mRNA protects HT1080 fibrosarcoma cells from ferroptosis based on reduced iron abundance and lipid peroxidation [77]. Furthermore, in OS cell lines and tissues, *miR-144-3p* expression is reduced while *ZEB1* expression is elevated, and it was found that *miR-144-3p* negatively regulates *ZEB1* expression [78]. The overexpression of *miR-144-3p* has the potential to decrease *ZEB1* expression, induce ferroptosis, and inhibit the growth, migration, and invasion of OS cells. The same study revealed that *ZEB1* and *miR-144-3p* in exosomes can control OS growth by altering the ferroptosis process [78]. Another micro-RNA, *miR-1287-5p* is downregulated in human OS, and its overexpression induces ferroptosis, most likely via the inhibition of GPX4 [79]. 

Molecular analyses of lipid peroxidation markers could provide critical insights into OS development and progression and can also serve as prognostic implications for disease outcomes and survival rates. However, more studies are needed in order to understand the possible clinical application of these findings and validate their impact on disease risk and progression [30,80]. 

## 4. Lipid Peroxidation-Related Redox Signaling in OS

### 4.1. Cysteine-Containing Proteins

Cys residues in proteins and peptides are very important for redox signaling, as the free thiol group is very sensitive to oxidation and can be selectively and reversibly oxidized. In addition, the oxidation of Cys can act via disulfide bridges as a link between redox-sensitive proteins, leading to their aggregation and eventual inactivation [26,81].

#### 4.1.1. GCL/GSH/GPX-GST Pathway

GSH is a tripeptide (Glu-Cys-Gly) that is produced in large quantities in the cells and acts as an antioxidant and regulator of the redox system. The GSH system, which includes glutamate-cysteine ligase (GCL), glutathione synthetase (GSS), reduced GSH, oxidized GSH, glutathione peroxidase (GPX), glutathione reductase (GR), nicotinamide adenine dinucleotide phosphate (NADPH), and GST, is another important regulator of cellular redox homeostasis [23,35,74,75]. The products of lipid peroxidation such as acrolein and 4-HNE are able to induce glutamate cysteine ligase (GCL) expression [82,83]. The α,β-unsaturated 15-deoxy-Δ^12,14^-prostaglandin-J_2_ (15d-PGJ_2_) is also able to upregulate GCLc in MG-63 OS cells [84].

4-HNE interacts with Cys residues on both GCLc and GCLm and could promote or prevent aggregation and activation of the enzyme depending on the order of the reaction. However, it can also bind directly to free GCLc and increase its catalytic activity [85]. In GCLc, Cys553 is activated by 4-HNE, but in the holoenzyme it is masked and 4-HNE cannot reach it, whereas Cys35 is exposed by GCLm [85,86].

GCL is elevated in many cancers [87] and this contributes to resistance to chemotherapy [88]. The same is true for OS, where an increased expression of GCL is found [84]. 

Cys for GSH synthesis is imported via the cystine/glutamate antiporter SLC7A11, which is overexpressed in human cancers [82]. Cancer cells must undergo metabolic reprogramming to provide high levels of glutamine to SLC7A11 to supply sufficient GSH for its function [89]. The overexpression of GPX4 is found in cancers with poor prognosis [90] together with SLC7A11 [91,92]. The same is true for OS, which shows an increased expression of SLC7A11 [71]. In fact, the overexpression of SLC7A11 may even suppress ferroptosis and promote cancer growth [89]. The tumor suppressor *P53* is often mutated or inactive in OS, but when active, SLC7A11 expression is downregulated [89,93]. The influx of Cys into cells could also be prevented by glutaminase inhibitors that block the conversion of glutamine to glutamate, thereby reducing the available glutamate pool for SLC7A11 [89,94]. Targeting ferroptosis can help in the treatment of chemotherapy-resistant OS [95]. High levels of GPX4, GSH, and SLC7A11 make OS resistant to ferroptosis, so targeting these factors may help in therapy [93,95]. 

GPX4 is active against phospholipid hydroperoxides (PLOOHs) and related cytotoxic compounds and reduces them with the help of GSH [96]. It is the only GPX that can catalyze the reduction of 7α-cholesterol-hydroperoxide (Chol 7α-OOH) [97]. In drug-resistant OS cell lines, GPX4 increased significantly in response to cisplatin; these OS cell lines had lower levels of ROS, lipid peroxides, and MDA compared to the original line [98].

GST protects cells from damage by conjugating xenobiotics, carcinogens, reactive aldehydes, quinones, epoxides, and hydroperoxides, which are formed as secondary metabolites of ROS. These include, for example, 4-HNE, acrolein, and 15-deoxy-delta(12,14)-prostaglandin J2 (15d-PGJ2) [99,100]. The GSTA4-4 isoform has a higher affinity for 4-HNE than for other substrates [101]. There is no data on the expression of GSTA4-4 in OS. However, there is a significant correlation between polymorphisms in *GSTT1* or *GSTM3* and the prognosis in OS. The *GSTM1* null genotype is correlated with relapse, while the GSTM3*B allele is associated with better overall survival. The GSTM3*B/GSTM1 or GSTM3*B/GSTT1 genotype that is present in metastatic patients at the time of diagnosis is associated with better survival [102].

#### 4.1.2. Peroxiredoxins and Thioredoxins

Peroxiredoxins (PRDXs) are responsible for maintaining redox homeostasis by degrading hydrogen peroxide and lipid peroxides [103]. They are the most abundant antioxidant proteins, containing a Cys residue at the catalytic site [104]. High concentrations of substrates such as LOOH can oxidize and inactivate PRDX3 and PRDX5 in the mitochondria [105,106]. This process is reversible, and the PRDXs are recycled by TRXR in an NADPH-dependent reaction or by sulfiredoxin (SRX) [26]. PRDXs act as both tumor suppressors and promoters, as the disruption of PRDX expression can lead to an increased incidence of cancer [107,108], but once developed, cancers increase PRDX expression. This expression contributes to resistance to chemotherapy and radiotherapy [109], so PRDX inhibitors are explored as therapeutic agents in various cancer models [107]. PRDX6 is present in various cancers and can reduce H_2_O_2_, short-chain hydroperoxides, and PLOOH in the cell membrane with the help of GSH, thus restoring cancer cell membranes [110]. Various reactive aldehydes have been tested to interfere with its activity: 4-HNE, MDA, acrolein, and 4-oxononenal (4-ONE), but apart from conformational changes and extensive cross-linking with multiple modification sites, they were unable, under pathophysiological concentrations, to interfere with its activity. The active site Cys47 remained protected due to conformational changes [111,112]. So far, two PRDXs have been described in OS: PRDX2, which contributes to resistance to chemotherapy, malignancy, and poor prognosis [113], and PRDX1 with conflicting results [114,115]. Some data suggest that the overexpression of PRDX1 and SRX1 may protect the liver from CCl4-induced toxicity, and reduce the amount of 4-HNE [116].

The thioredoxin (TRX) system, which includes TRX, thioredoxin reductase (TRXR), NADPH, and thioredoxin interacting protein (TXNIP), is another important regulator of cellular redox homeostasis [35]. TRX maintains the redox environment in cells by reducing disulfide bonds in proteins (such as PRDX). In its catalytic center, TRX has a pair of redox-active Cys residues that are oxidized during activity and recycled and reduced by the reducing enzyme TRX reductase (TRXR) [81]. The expression of TRX and TRXR is associated with the progression and metastasis of various types of cancer [117]. This is also true for OS, where a high expression of TRX is a factor in metastasis and a poor prognosis [118]. The oxidation of TRX prevents its binding to apoptosis signal-regulating kinase 1 (ASK1), and the free protein kinase ASK1 is activated and can induce apoptosis. This demonstrates the importance of TRX in the regulation of ROS-mediated apoptosis [81]. Acrolein interacts with the active groups Cys32 and Cys35 of TRX as well as with Cys59/Cys64 and Cys497 of TRXR and inactivates it, which in the case of TRXR is an irreversible reaction [119]. 4-HNE interacts with the residues Cys73 and Cys32 of TRX [120].

Nucleoredoxin (NRX) belongs to the TRX family, but unlike TRX, it appears to be a multifunctional enzyme that probably acts as an oxidoreductase. It also contains a pair of Cys in its catalytic site, Cys205 and Cys208, which are essential for its activity [120,121]. NRX deficiency increases the cellular levels of NADPH and GSH [122]. In this context, a downregulation of NRX is observed in carcinomas compared to normal tissues [123]. It is assumed that NRX plays a certain role in cellular differentiation via the Wingless and Int-1 (WNT) signaling pathways [124]. NRX is important during bone development and for bone homeostasis and likely acts by inhibiting the WNT signaling pathway [125]. There is no data on the involvement of NRX in OS or the interaction of lipid peroxidation products with NRX.

#### 4.1.3. Redox Sensitive Proteins

Several other proteins have Cys in the active site that is regulated by oxidation and can be modified by lipid peroxidation products and reactive aldehydes. Protein tyrosine phosphatases (PTPs) are a family of enzymes that catalyze the dephosphorylation of phosphotyrosine in proteins. The catalytic site of PTPs contains a Cys residue that serves as a transient receptor for phosphate. In the case of oxidation, the reaction cannot take place, the enzyme is blocked, and the phosphotyrosines are increased. These reactions are reversible, and the functions of the PTPs could be restored [81]. One of these PTPs is the phosphatase and tensin homolog (PTEN). When Cys is oxidized in the catalytic site, it forms a disulfide bond to protect itself from irreversible oxidation, and with the help of reducing enzymes such as TRX, disulfide bonds can be reduced [81]. PTP1B is involved in the regulation of insulin signaling and also contains Cys in the catalytic site, which is oxidatively regulated [81]. MAP kinase phosphatase (MKP) is involved in the stress signaling pathway by downregulating c-Jun N-terminal kinase (JNK). When oxidized, MKP activity is inhibited, and JNK activation is increased. The oxidation of MKP results in the formation of high molecular weight protein complexes that are degraded by proteasomes [81]. Essential glycolytic enzymes such as glyceraldehyde-3-phosphate dehydrogenase (GAPDH) and the cancer cell-specific pyruvate kinase isozyme M2 are inactivated by the oxidation [126,127] of Cys residues [126,128,129]. 

In addition, aldehydes derived from lipid peroxidation, such as 4-HNE, can modify a variety of proteins and alter their structure and function due to their high reactivity [48,130,131,132]. Some modifications of redox-sensitive proteins have biological relevance, as recently reviewed [50]. 

### 4.2. NADP^+^/NADPH and NAD^+^/NADH Redox Systems

The nicotinamide adenine dinucleotide phosphate NADP^+^/NADPH and the nicotinamide adenine dinucleotide NAD^+^/NADH are crucial for the maintenance of cellular redox homeostasis and cellular energy metabolism. The NADP^+^/NADPH system is involved in the maintenance of redox balance, NADPH oxidase (NOX) activity, synthesis of fatty acids, amino acids, nucleotides, and steroids, while the NAD^+^/NADH system is involved in the citric acid cycle, glycolysis, and oxidative phosphorylation. Both coenzymes are involved in cellular oxidation/reduction reactions, with NAD^+^/NADH being primarily responsible for oxidation reactions and NADP^+^/NADPH for reduction reactions. The reason for this is the difference in their redox potential; the NADP^+^/NADPH pair is more electronegative than the NAD^+^/NADH pair [133,134,135]. The amount of NAD^+^/NADH is about 10 times higher than the concentration of NADP^+^/NADPH in the same cells [136].

#### 4.2.1. NADP^+^/NADPH

To protect themselves from oxidative stress and maintain redox homeostasis, cells have developed complex enzymatic antioxidant defense systems that depend on the key molecule NADPH, which provides electrons to recycle PRDX, TRX, and GSH. The main source of NADPH in the cell is the pentose phosphate pathway (PPP) [137] with the rate-limiting enzyme glucose-6-phosphate dehydrogenase (G6PD) [138]. It is also produced secondarily by malic enzyme (ME), isocitrate dehydrogenase (IDH), nicotinamide nucleotide transhydrogenase (NNT) [139,140,141,142,143,144], and NAD+ kinase (NADK) [145].

G6PD is a cytosolic enzyme that catalyzes the reaction between glucose-6-phosphate and NADP+, during which NADPH is formed. The upregulation of G6PD is found in various cancers, and its inhibition has a cytotoxic effect on cancer cells [146]. G6PD-deficient cells exhibit higher levels of the lipid peroxidation product MDA when exposed to oxidizing agents [147]. OS cells also show increased levels of G6PD [148]. NADP^+^ is required for the enzymatic activity of G6PD, whereas NADPH downregulates it. This can be prevented by an increase in GSSG or a higher consumption of NADPH in cancer cells [143].

ME is an oxidoreductase that converts malate to pyruvate through the formation of NADPH. Cytosolic ME1 and mitochondrial ME2 appear to be the most important isoforms. Their overexpression is associated with cancer growth and a poor prognosis. The silencing of both ME1 and ME2 leads to a reduction in NADPH production, increased ROS formation, and increased sensitivity to chemotherapy [143,149]. There are two MEs that are expressed in osteoblasts. ME2 (NADH-dependent) is specifically upregulated during osteoblast differentiation and its inhibition impairs osteoblast proliferation and differentiation, whereas ME1 (NADPH-dependent) remains unchanged [150]. ME1 is upregulated in OS and is a risk factor for OS progression [151].

IDH generates NADPH from NADP+ by catalyzing the oxidative decarboxylation of isocitrate to α-ketoglutarate (α-KG). There are two forms of the enzyme: IDH1 in the cytosol and in the peroxisomes, and IDH2 in the mitochondria. IDH1 is overexpressed in many types of cancer and is closely associated with poor prognosis [143]. The knockdown of IDH1 leads to a decrease in NADPH and α-KG and an increase in ROS, leading to cancer cell apoptosis. This also increases the sensitivity of cancer cells to chemotherapy and radiotherapy. IDH2 is also overexpressed in cancer, lowering ROS and increasing the growth of cancer cells [152]. IDH1 and IDH2 are frequently mutated in cancer, which alters their activity so instead of generating NADPH, NADPH is consumed by the reduction of α-KG to generate 2-HG [153]. 4-HNE is able to decrease the activity of IDH2 in cardiomyocytes and reduce NADPH production. All these effects can be prevented by the addition of GSH [154]. Purified IDH is inactivated by MDA and 4-HNE, but at rather high mM concentrations, whereas LOOH acts at µM concentrations. Binding to IDH is confirmed by ESI-MS for 14 residues [155]. Since Lys212, His315, and Cys387 are essential for the activity of IDH2, an interaction with Hys and Cys is to be expected [85,86].

NNT is an enzyme of the inner mitochondrial membrane that uses the energy of the proton gradient to generate NADPH. This NADPH is then used within the mitochondria for biosynthesis and to maintain reduced GSH. NNT expression, as analyzed using the Cancer Genome Atlas cohort and Gene Expression Omnibus datasets, is lower in HCC patients than in controls [156]. No data are available on NNT expression in OS.

NADK consists of two enzymes: cytosolic NADK1 and mitochondrial NADK2, which phosphorylate NAD+ and produce NADP+ [145]. In some cancers, NADK contributes to carcinogenesis, particularly through mutations that can increase its activity and cause a greater increase in NADPH production, which then serves as an antioxidant defense. Silencing or overexpressing NADK1 has only a modest effect on ROS levels, but NADK2 may play some role by protecting mitochondria from ROS [145]. There is no data on the expression of NADK in OS and no data on its interaction with lipid peroxidation products.

Although NADPH is used for reduction reactions, it is also a substrate for the generation of free radicals by NOX1-5. These enzymes generate small amounts of ROS locally for redox-sensitive signaling that supports cancer progression, such as the stimulation of oncogenes (*SRC, RAS*) or inactivation of tumor suppressors (P53, PTEN) [143,157]. NOX is important for bone homeostasis and differentiation. While NOX4 produces small amounts of H_2_O_2_ for redox signaling and is involved in osteoblast differentiation, NOX1 and NOX2 produce O_2_ˉ mainly in osteoclasts and probably play a role in bone remodeling and are even involved in OS migration. NOX4 interferes with signaling cascades such as KEAP/NRF2 or mediates the stability of HIF1 by controlling the activity of prolyl hydroxylases [158]. NOX2 is overexpressed in several human cancers, including OS, and is associated with a poor prognosis [159]. In all OS cell lines examined, NOX2 and NOX4 are expressed, whereas NOX1 and NOX3 are hardly or not at all detected. Both NOX2 and NOX4 silencing lead to a reduction of ROS, reduced viability of OS, and apoptosis [160]. The overexpression of NOX4 decreased five mitochondrial oxidative phosphorylation (OXPHOS) enzyme complexes and ATP production and increased 4-HNE, MDA, and the accumulation of iron [161]. The disruption of NOX1 and NOX4 (especially NOX1) in cardiomyocytes makes them more sensitive to the product of lipid peroxidation, acrolein. Alkylating agents that act as NOX inhibitors bind to one of the four Cys residues in the active site of the enzyme [162]. Since 4-HNE and acrolein are also alkylating agents, it is possible to interfere with NOX activity [163].

NADPH also serves as a cofactor for antioxidant enzymes, some of which have already been mentioned in the sections above: GR, GPX, TRX, TRXR, etc. [26]. Catalase (CAT) is the antioxidant enzyme with a heme b in the active site and NADPH as a cofactor that breaks down H_2_O_2_ into O_2_ and H_2_O. It can handle high concentrations of H_2_O_2_. In tumors, CAT is often downregulated, and low CAT correlates with high H_2_O_2_ production. There is evidence that CAT is involved in cell signaling, proliferation, differentiation, and apoptosis. It appears that in some cancer cells, an increased CAT expression is a response to chemotherapy or radiotherapy and protects them from ROS [164,165]. In several human cell lines, CAT activity increased in response to the lipid peroxides 13-hydroperoxy-9,11-octadecadienoic acid (13-HPODE), 13-hydroxy-9,11-octadecadienoic acid (13-HODE), or H_2_O_2_ [166].

There is a new experimental approach in OS therapy using metal nanozymes that mimic CAT activity and break down H_2_O_2_ to O_2_. The idea behind this is to generate O_2_ in the cancer to combat hypoxia of the cancer tissue. Hypoxia leads to the expression of genes that are involved in the stress response and provide protection to cancer cells. Overcoming hypoxia could make the cancer more sensitive to anticancer therapy [167].

#### 4.2.2. NAD^+^/NADH

The NAD^+^/NADH redox couple is a regulator of cellular energy metabolism, and its precise balance is necessary for normal physiological functions [168]. De novo synthesis of NADH begins with the transport of tryptophan (Trp) by the SLC6A19 transporter, followed by a sequence of enzymatic processes involving indoleamine 2,3-dioxygenase (IDO); tryptophan 2,3-dioxygenase (TDO); arylformamidase; kynurenine 3-monooxygenase, kynureninase, and 3-hydroxyanthranilate 3,4-dioxygenase; ACMS decarboxylase; quinolinate phosphoribosyl transferase; and nicotinamide mononucleotide adenylyl transferases [168]. In the final step, NAD^+^ synthase uses glutamine and ATP to produce NAD^+^. The second pathway of synthesis is via the salvage pathway, in which NADH precursors are recycled [169]. 

Trp exposure leads to an increase in the lipid peroxidation products 4-HNE and MDA in tested subjects, in parallel with an increase in kynurenines. The oxidative stress may result from the generation of quinolinic acid, 3-hydroxykynurenine, and 3-hydroxyanthranilic acid, all of which are known to have the ability to generate ROS [170]. 

IDO is a heme oxygenase and the rate-limiting enzyme for NAD^+^ synthesis [171]. It contains the residues Cys99, Cys126, Cys143, Cys173, and Cys220, which could be modified by H_2_O_2_ blocking its activity. The activity of the enzyme could be rescued by TRX [172]. Although reactive aldehydes and LOOH could block the activity of the enzyme by binding to Cys residues, there is no data on this. The only experiment that has been performed studied the colocalization of 4-HNE and IDO in pre-eclamptic human placenta, and it was not found [173]. IDO is expressed in most of the OS [174] and acts as an immunosuppressive enzyme that helps several malignant tumors, including OS, escape the immune system. A high expression of IDO is associated with a poor prognosis and metastasis in OS patients [175]. In addition, the expression of IDO and programmed death ligand 1 (PD-1) is higher in OS who have received neoadjuvant chemotherapy, suggesting an adaptive mechanism to therapy [176,177]. 

TDO is heme dioxygenase and contains conserved His72 at its active site, and mutations can decrease but not prevent its activity. It participates in the initial and rate-limiting steps of the kynurenine pathway of Trp metabolism. TDO is special because it is reductively reactivated by an oxidant, H_2_O_2_, in the presence of Trp and also possesses CAT-like activity [171,177]. It is expressed in many tumors and acts as an immunosuppressant, preventing tumor rejection [33,35]. There is no data on the effects of lipid peroxidation products on expression and TDO in OS, but in human glioma cell lines, PGE2 can upregulate TDO2 [178]. The expression of TDO2 is downregulated by increased HIF1α during hypoxia, whereas knocking out HIF1α restores the expression of TDO2. In addition, overall Trp metabolism is downregulated in part by HIF1α during hypoxia [179]. 

### 4.3. Redox Sensitive Signaling in OS

#### 4.3.1. KEAP1/NRF2 Signaling Pathway

The main defense mechanism against stress conditions such as oxidative stress and xenobiotics is the inducible KEAP1/NRF2 (Kelch-like ECH-associated protein 1) pathway, which tightly regulates redox homeostasis [180,181,182,183]. The KEAP1 repressor binds NRF2, which is then ubiquitinated by the Keap1-Cullin3 E3 ubiquitin ligase and degraded by proteasomes. Under stress conditions, NRF2 is released from KEAP1 and binds to the antioxidant response element (ARE) of the target genes, leading to their activation [184,185,186].

There are many cellular processes that are influenced by NRF2, such as the antioxidant response, regulation of metabolism, and mitochondrial function. Some of the genes are responsible for protecting cells from ROS: heme oxygenase 1 (HMOX1), GCLc, GCLm*,* GSS, GST, GR, SOD1, CAT, TRX, TXNRD1, SRX, PRDX1, drug transport (MRP gene family), quinone 1 (NQO1), NAD(P)H dehydrogenase, etc. [185,187,188,189].

The release of NRF2 from KEAP-1 is mediated by the modification of a specific thiol group in KEAP-1 by electrophiles. Those electrophiles are reviewed in [190] and include lipid peroxidation products and other electrophilic products of metabolism. 

Current data suggest that Cys151, Cys273, and Cys288 are the functionally important Keap1 Cys residues. While Cys273 and Cys288 can be important in binding to Nrf2, Cys151 seems to be important in directing NRF2 to ubiquitination and degradation [191]. Cys151 and Cys288 of KEAP-1 are attacked by electrophiles such as 4-HNE and acrolein, but Cys288 seems to be a specific alkenal stress sensor for 4-HNE and acrolein [192]. Other electrophiles could also be important in modifications of KEAP-1, such as LOOH 15d-PGJ_2_ by binding to Cys273 and Cys288 residues [191].

**Figure 2:**
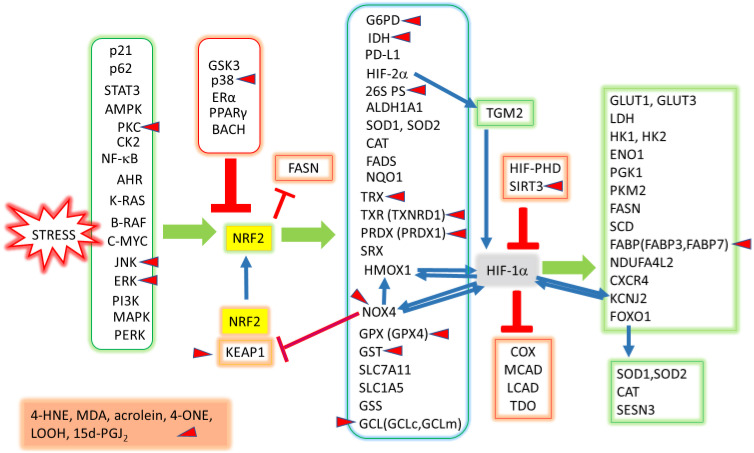
Interference of lipid peroxidation products 4-HNE, MDA, acrolein, 4-ONE, LOOH, and 15d-PGJ2 with redox-sensitive stress signals. The occurrence of stress leads to the activation of redox-sensitive signalling pathways that serve cellular protection. The most important regulatory mechanisms for protection and adaptation are the NRF2 and HIF1 signalling pathways together with their target molecules. Green arrows represent signalling pathways induced by the stress response, i.e., increased activity or expression of signalling molecules. The red T stands for suppressed signalling pathways, with the flat top of the T representing inhibited activity or reduced expression of signalling molecules. Blue arrows indicate the direction of positive regulation between individual signalling molecules, with double-sided arrows representing bidirectional positive regulation. The red triangles represent confirmed direct interactions between lipid peroxidation products and signalling molecules involved in NRF2 and HIF1 signalling pathways. This indicates that lipid peroxidation products interfere with and modulate cellular stress signalling. The following works form the basis for the creation of this scheme [35,48,193].

Redox signaling involving lipid peroxidation products is shown in Figure 2.

Mechanisms enabling NRF2 activation are reviewed in [186]. There are several oncogenes and transcription factors that are involved in NRF2 activation. Those include endogenous oncogenes *K-RAS*, *B-RAF*, and *MYC* [187]; aryl hydrocarbon receptor (AHR) [194]; nuclear factor (NF)-κB; extracellular signal-regulated protein kinases (ERK) [195]; c-jun JNK [195], PI3K-AKT, and p38 MAPK [196,197]; protein kinase C (PKC) [198], casein kinase 2 (CK2) [199]; and protein kinase R (PKR)-like endoplasmic reticulum kinase (PERK) [200]. Some of the transcription factors could directly influence NRF2 expression by binding to the NRF2 promoter [201]. The NRF2 promotor includes two regions: ARE-like, XRE-like (for AHR), and it also contains the Nf-κB binding domain, allowing direct activation by NF-κB [202], MYC, and JUN binding sites [187] while KRAS may bind to the TRE response element. Besides this, PKC and CK2 may directly prevent the interaction of KEAP1 and NRF2 by phosphorylating NRF2 [201]. There are also negative regulators of NRF2. Some of those are peroxisome proliferator-activated receptor gamma (PPARγ) [203] and estrogen receptor α (ERα) [204], which can bind to NRF2 and suppress NRF2 activity, or p38 and GSK3 can decrease NRF2 stability by phosphorylation [196].

The activation of NRF2 protects cells from carcinogens and mutagens and has protective roles against tumor initiation [186], but in already established tumors, increased NRF2 activity is found, enhancing ROS detoxification and protecting tumor cells from chemotherapeutics, and is associated with a poor prognosis [187,202,205,206].

Low doses of 4-HNE could upregulate NRF2 and consequently upregulate the cellular antioxidative system by enhancing the expression of GCL and the Xc(-) subunit of SLC7A11 and consequently increasing the GSH level. Those cells then become resistant to cytotoxic concentrations of either reactive aldehydes or ROS [207].

NRF2 is important in the metabolic reprogramming of cancer cells. NRF2 not only affects the antioxidant response, but also glycolysis, PPP, amino acid metabolism, and glutaminolysis, resulting in increased amounts of products directed into the TCA cycle and the mitochondrial respiratory chain [197].

The glutamine transporter SLC1A5 is activated by NRF2, ensuring glutamine for cellular processes [208]. Related to this, GLS2 and GPT2 are activated by NRF2, and they direct glutamine into the production of glutamate, α-ketoglutarate, GSH, etc., to create building blocks for cancer cells [197]. All enzymes necessary for the production of GSH—GCLc, GCLm, and GSS are controlled by NRF2 [189].

Several key enzymes in lipid metabolism are activated by NRF2, including fatty acid desaturase 1 (FADS1), elongation of very long chain fatty acids protein 7 (ELOVL7), acyl-CoA thioesterase 7 (ACOT7), acyl-CoA synthetase short-chain family member 2 (ACSS2), acyl-Coenzyme A dehydrogenase family member 12 (ACAD12), and acyl-Coenzyme A dehydrogenase family member 10 (ACAD10) [209]. FADS contain conserved His residue in the active site and this His is important for fatty acid elongation by ELOVL [210]. ACOT7 does not contain any amino acid residue which could be attacked by electrophile [211], but ACSS2 contains Lys residue at active site.

Damaged proteins are degraded by the 26S proteasome, which consists of a 20S core and a 19S regulatory subunit. NRF2 regulates the expression of genes in multiple subunits of the 20S proteasome and the 19S proteasome [212]. Proteasomal inhibitors could be helpful in treating OS [213].

The inducible form of heme oxygenase HMOX-1 is expressed at a low level under normal basal conditions and increases under pathological conditions. In tumors, it increases vascularization, thus promoting tumor growth and their metastatic potential. It acts as an antioxidant enzyme and conveys an antiapoptotic effect through the p38 MAPK pathway, the NF-κB/PI3K/AKT pathway, and others [214,215]. It is found to be increased in patients with OS [216] and an increased expression in OS cells protects them from ferroptosis and chemotherapy [217]. The inhibition of HMOX-1 causes iron accumulation and the formation of ROS and LOOH [218]. HMOX-1 lacks Cys residues at its active site, so lipid peroxidation products probably cannot directly modulate HMOX-1 activity. However, it is noted that HMOX-1 could be palmitoylated [219].

When compared to adjacent normal tissues, malignant and benign bone tumors have a significantly lower expression of proapoptotic *BAX*, caspase-8, caspase-9, and the detoxification enzyme GPX4. A high expression of *BCL-2*, which could act as an anti-apoptotic mediator, was detected in high-grade and metastatic OS, together with the high transferrin receptor TFR1 [220].

The signal transducer and activator of transcription 3 (STAT3) reacts to stress and is usually activated in many cancers, leading to cancer progression. It activates the cellular antioxidative response by upregulating NRF2 and, consequently, GPX4. In OS cell lines resistant to cisplatin due to chronic exposure, STAT3, NRF2, and GPX4 are significantly increased. Those cells have lower levels of ROS, lipid peroxides, MDA, and apoptosis when compared to the original line. The chronic use of cisplatin induces the generation of drug-resistant OS cells by inhibiting ferroptosis through the STAT3/NRF2/GPX4 signal transduction pathway [98].

The metastatic potential of OS cells is correlated with aldehyde dehydrogenase 1A1 (ALDH1A1) activity [221] and overexpression is associated with poor cancer prognosis in some cancers [222] and resistance to doxorubicin and cisplatin therapy in vitro [223]. This NAD(P)^+^-dependent enzyme is upregulated in cancer cells by NRF2 [224] and catalyzes the detoxification of products of lipid peroxidation, which are reactive aldehydes such as 4-HNE, MDA [225], and 4-ONE [226]. Another ALDH isoenzyme, ALDH2 is found to be reversibly modified by 4-HNE at low concentrations, while irreversibly modified with high 4-HNE and low 4-ONE concentrations by affecting Cys residue at the enzyme active site [226]. Although there is no data on ALDH1A1, Cys303 is present in the active site, and due to a much more open and broader active site than ALDH2 active site there is no steric disturbance, and the ALDH1A1 active site is accessible to reactive aldehydes [227].

#### 4.3.2. HIF-1α

The cellular response to hypoxia is based on the activation of multiple genes involved in many biological processes. Among them, hypoxia-inducible factor (HIF) is the main regulator of the response to hypoxia in both healthy and cancer tissue [228,229,230].

The HIF family consists of HIF-1α, HIF-2α, HIF-3α, and HIF-1β. The heterodimeric structure is composed of the O_2_-sensitive α subunit and β subunit, and the assembly process activates HIF. In normoxic conditions, the HIF-1α subunit is hydroxylated by HIF prolyl-hydroxylases (PHD) and degraded by the ubiquitin-proteasome system. Under hypoxic conditions, HIF-PHD activity is inhibited, and HIF-1α accumulates in the cell, leading to its activation. HIF-1α conducts cellular response to hypoxia by regulating genes containing specific hypoxia response elements (HREs) by binding to them [228,231,232,233].

Hypoxia-inducible factor is an important modulator of cancer metabolic reprogramming in hypoxic conditions. Cancer cells have a high need for glucose, and different genes involved in glycolysis are upregulated under HIF-1α transcriptional control: glucose transporters (GLUT1, GLUT3), lactate dehydrogenase (LDH), glycolytic enzymes hexokinase 1 and 2 (HK1, HK2), enolase 1 (ENO1), phosphoglycerate kinase 1 (PGK1), pyruvate kinase M2 (PKM2), etc. [233,234,235].

Under normoxic conditions, pyruvate dehydrogenase (PDH) transforms pyruvate with cofactors NAD^+^ and coenzyme A into acetyl-CoA and NADH, which may be further used in the citric cycle. But in hypoxia, HIF-1α induces pyruvate dehydrogenase kinase 1 (PDK-1) and PDH is inactivated, leading to pyruvate metabolism by LDH and the formation of lactate [236]. This shifts metabolism from OXPHOS to anaerobic glycolysis [237]. The produced lactate stabilizes HIF-1α and creates an acidic condition in cancer cells environment, and promotes cancer invasion and metastasis [233,238].

Products created during the process of glycolysis are used for nucleotide and lipid synthesis [239]. Cancer cells are in high demand for fatty acids, so HIF-1α upregulates fatty acid (FA)-binding proteins FABP3 and FABP7, increasing FA transport in cells [240]. FABP7 binds long-chain polyunsaturated FA (PUFA), allowing uptake and intracellular trafficking [241]. However, in ischemic conditions, those FABPs cause damage due to an increased transport of FA and consequently the occurrence of lipid peroxidation products such as 4-HNE [242]. Although there is no data about the interaction between lipid peroxidation products and FABP3 and FAB7, other FABPs are explored as targets. 4-HNE binding can have different consequences; so, FABP4 possesses Cys117, which is the target for 4-HNE, and its binding impairs protein affinity for fatty acids [243], but FABP5 Cys-120 modification by 4-HNE forms a more stable protein [244].

HIF-1α also suppresses mitochondrial FA oxidation by inhibiting medium-chain Acyl-CoA dehydrogenase (MCAD) and long-chain Acyl-CoA dehydrogenase (LCAD). This is done by suppressing *c-MYC*, a transcriptional coactivator of PGC-1β, required for MCAD and LCAD. HIF-1α also promotes the expression of fatty acid synthase (FASN) to trigger FA synthesis and stearoyl-CoA desaturase (SCD) to generate unsaturated FA [235]. The inhibition of FA oxidation contributes to redox homeostasis in hypoxia, decreased ROS production, the suppression of the PTEN pathway, and the promotion of cancer cell proliferation [245].

Due to their central role in cellular energy metabolism, mitochondria are the main consumers of oxygen in the cell. Mitochondrial adaptation to hypoxia includes modifications in the respiratory chain, decreased OXPHOS, the tricarboxylic acid (TCA) cycle, and β-oxidation. Moreover, mitochondrial TCA cycle intermediates participate in modulating HIF activity [246].

Complex IV (cytochrome oxidase, COX) is affected in hypoxia by HIF-1α. HIF-1α induces the expression of COX4I2 and the mitochondrial LON protease, leading to COX4I1 targeting by LON for proteasomal degradation. Due to these processes, COX4I2 replaces COX4I1 in complex IV, which improves the activity of complex IV and produces less ROS. Complex I activity is suppressed by HIF-1α through an increased expression of the mitochondrial NDUFA4L2 encoding NADH dehydrogenase (ubiquinone) 1/subcomplex subunit 4-like 2, which suppresses Complex I activity, leading to a decreased ROS production [228,247].

HIF-1α can be stabilized by ROS generated by the NADPH oxidase system, in particular NOX. The expression of NOX1 increases the expression of both HIF-1α and HMOX1 [248]. NOX4 interferes with the signaling cascades of KEAP/NRF2, increasing free NRF2 [249] or increasing the expression of HIF-1α protein [250]. This pathway seems bidirectional, as HIF-1α is also able to induce NOX4 [251].

*NRF2*-silencing decreased HIF-1α accumulation in hypoxic cancer cells. HIF-1α dysregulation in *NRF2*-silenced cancer cells was associated with short non-coding miRNA *miR-181c* elevation. Also, in *NRF2*-silenced cells, hypoxia is not able to completely restore the increased levels of metabolites involved in glycolysis and the activated PPP pathway [252].

Chronic hypoxia is able to induce HIF-2α through the NRF2 pathway. *NRF2*-silencing prevents HIF-2α accumulation, together with the inhibition of cancer migration and spheroid growth. This process is associated with an increased expression of the short non-coding miRNA *miR-181a-2-3p* in NRF2-silenced cells [253].

HMOX1 is also a downstream target of the transcription factor HIF-1α. HMOX1 acts both downstream and upstream of HIF-1α, and the stabilization of HIF-1α contributes to protection against ischemic injury [254].

HIF-1α is overexpressed in many carcinomas, including OS. There are several pathways through which HIF-1α affects OS progression. In OS tissue, there is the expression of a high level of HIF-1α together with the chemokine receptor CXCR4. This receptor contributes to the metastasis of many cancers. Under hypoxic conditions, OS migration and invasion are enhanced, but blocking HIF-1α or CXCR4 decreases them. By blocking HIF-1α, the expression of CXCR4 was blocked, showing that hypoxia induced CXCR4 expression through HIF-1α [255].

Mitochondrial NDUFA4L2 protein is upregulated under hypoxic conditions. NDUFA4L2 is a component of the electron transport chain complex I and decreases ROS production. HIF-1α inhibition decreases the NDUFA4L2 protein. The downregulation of NDUFA4L2 has a direct effect on the apoptosis of OS cells. In OS cells, it promotes migration, invasion, proliferation, and the epithelial–mesenchymal transition. HIF-2α does not regulate NDUFA4L2 expression [256].

Potassium inwardly rectifying channel subfamily J member 2 (KCNJ2) is overexpressed in advanced-stage OS tissues in cells with high metastatic potential and is associated with a shorter survival rate in patients with OS. KCNJ2-inhibition prevents OS metastasis, whereas KCNJ2-increase increases it. KCNJ2 binds to HIF-1α and inhibits its ubiquitination, thus increasing the amount of HIF-1α. On the other hand, HIF-1α binds directly to the *KCNJ2* promoter and increases its transcription under hypoxic conditions [257].

The upstream regulation of HIF-1α accumulation is also regulated by transglutaminase 2 (TGM2), which creates a status called pseudohypoxia [258]. Enhanced TGM2 expression in metastatic OS contributes to the migratory and invasive properties of OS. TGMs metabolize glutamine into glutamate and enable substrates for GSH synthesis and to drive cellular processes [259]. TGM2 is also responsible for the chemoresistance of OS cells to cisplatin [260].

Microarray data of OS and healthy tissue revealed an increased expression of HIF-1α, Forkhead box protein O1 (FOXO1), Acylphosphatase 1 (ACYP1), Peptidylprolyl Isomerase H (PPIH), Peptidylprolyl Isomerase E (PPIE), and Sestrin 1 (SESN1) and a decreased expression of *CYP-*related genes (cytochromes p450) in OS tissue. The link between the genes was tested in OS using *HIF-1α* silencing and significantly decreased CAT, SOD1, SOD2, NOX4, SESN3, FOXO1, PRDX1, and GPX1 was found, while significantly increased were subunits from the P-450 family (Cyp2c38, Cyp2c55, and Cyp2c29), COX2, and arachidonate 12-lipoxygenase, together with an increased ROS production. The overexpression of HIF-1α increased FOXO1 expression by targeting HRE in the promoter region of FOXO1, and over FOXO1 are induced SOD2, CAT, and SESN3, resulting in an antioxidative effect and the inhibition of ROS formation [261]. FOXO factors have been involved in regulating cell cycle progression and apoptosis, resistance to chemotherapy, and the detoxification of ROS [262], while 4-HNE was found to activate FOXO in bone cells [263].

LOOH and reactive aldehydes could interfere with HIF-1α signaling pathways. 4-HNE increased the growth of cancer cells and upregulated the level of HIF-1α through the inhibition of Sirtuin-3 (SIRT3). SIRT3 is a mitochondrial NAD+-dependent deacetylase and is reported to destabilize HIF-1α. 4-HNE could inhibit the deacetylase activity of SIRT3 by binding to its Zn-binding Cys280 residue [264,265]. In ischemic preconditioning of myocard, HIF-1α decreases tissue damage and prevents lipid peroxidation, as measured by the accumulation of 4-HNE by the activation of the ALDH2/SIRT3/HIF1α signaling pathway [266].

## 5. Lipid Peroxidation in OS Therapy

The relationship between specific drugs with oxidative stress and lipid peroxidation in the tumor has been established, and its possible application in tumor therapy is the focus of the research [267]. Cancer cells have altered metabolism and signaling pathways that make them more susceptible to oxidative stress-induced damage compared to normal cells. While lipid peroxidation is often associated with cellular damage and disease progression, certain lipid peroxidation products have shown potential therapeutic applications in cancer treatment. These lipid peroxidation products can exhibit anticancer effects through various mechanisms, including the induction of apoptosis, inhibition of cell proliferation, and modulation of certain signaling pathways (reviewed in [268]). For example, 4-HNE has been shown to possess anticancer properties by inducing apoptosis, inhibiting cell proliferation, and modulating signaling pathways involved in cell survival and metastasis. Additionally, 4-HNE can sensitize cancer cells to chemotherapy and radiation therapy. MDA, acrolein, isoprostanes, and oxidized phospholipids, which are all lipid peroxidation products, have shown potential anticancer effects by inhibiting cell proliferation and inducing apoptosis in cancer cells [268]. MDA-based conjugates have been explored as potential targeted drug delivery systems for cancer therapy. Still, further studies are needed to better understand their mechanisms of action and evaluate their efficacy and safety in clinical settings.

Since ferroptosis has emerged as a new hallmark of carcinogenesis, researchers are exploring the potential therapeutic applications of modulating ferroptosis, both to induce cell death in cancer cells and to prevent cell death in certain pathological conditions. Preclinical studies have demonstrated that inducing ferroptosis in OS cells can inhibit tumor growth and promote cell death. Various compounds and approaches targeting key regulators of ferroptosis pathways, such as GPX4 inhibitors or modulators of iron metabolism, have shown efficacy in preclinical and clinical models of OS [93] (Table 1).

Certain inorganic compounds, apart from iron, exhibit properties that can modulate lipid peroxidation, potentially impacting the viability and proliferation of cancer cells. For example, selenium particles have been found to have antioxidant properties, which can mitigate oxidative stress-induced damage in cancer cells. However, its effects on osteosarcoma cells have not yet been validated in clinical trials [269]. Other studies have shown that black phosphorus nanoparticles (BPNPs) possess intrinsic anticancer activity through various mechanisms, including the induction of oxidative stress and lipid peroxidation, selectively targeting cancer cells while sparing normal cells [270]. In osteosarcoma specifically, exfoliated black phosphorus (2D bP) can disrupt cancer cell metabolism and signaling pathways, ultimately inhibiting their proliferation and promoting cell death [271].

**Table 1:** Drugs targeting OS through the induction of ferroptosis.

Compound	OS Cell Lines	Signaling Pathways	Target Molecule	Mechanism of Action	References
Sulfasalazine	K7M2	X_c_^–^ system	SLC7A11	Accumulation of cytsolic and lipid ROS; inducing ferroptosis and lipid peroxidation by inhibiting SLC7A11; decreasing cellular GSH and GPX4 activity	[272]
Tirapazamine	HOS, U2OS, 143B	SLC7A11/GPX4	SLC7A11	Iron accumulation; increasing intracellular ROS; inducing ferroptosis under hypoxia by inhibiting the proliferation and migration of cells by downregulation of SLC7A11 and GPX4	[273,274]
Bavachin	HOS, MG63	STAT3/p53/SLC7A11	SLC7A11	Inducing ferroptosis by inhibiting STAT3 and enhancing p53, which inactivates SLC7A11; accumulation of ROS and MDA, GSH depletion, and the downregulation of GPX4 expression	[275]
KDM4A	143B, HOS	-	SLC7A11	GSH depletion, lipid peroxidation; inhibitingferroptosis by inducing SLC7A11 transcription by activating H3K9me3 demethylation	[276,277]
EF24	U2OS, Saos-2	HMOX-1/GPX4	GPX4	Lipid peroxidation, iron accumulation, and ROS production inducing ferroptosis by upregulating HMOX1 to suppress GPX4 expression	[278,279]
DFHHP	Saos-2	-	GPX4	Boosting the growth suppression of hypoxic OS by the induction of ferroptosis; GSH depletion, lipid peroxidation, iron accumulation, and ROS production	[280,281]
PEITC	143B, HOS, U2OS, K7M2, MG63	MAPK	GPX4	Inducing ferroptosis, apoptosis, and autophagy; altering iron metabolism, disturbing the redox balance, and activating the ROS-related MAPK signaling pathway; GSH depletion, lipid peroxidation, iron accumulation, and ROS production	[282,283]
STAT3 inhibitor	MG-63, Saos-2	STAT3/NRF2/GPX4	GPX4	Inhibiting STAT3/Nrf2/GPX4 signaling pathway; activating ferroptosis in OS cells and increase sensitivity to cisplatin; lipid peroxidation, iron accumulation, and ROS production	[98]
Butyrate	MNNG/HOS, U2OS	ATF3/SLC7A11	SLC7A11	Upregulating ATF3 expression and promoting erastin-induced GSH depletion; lipid ROS accumulation and enhancing erastin-induced ferroptosis	[284]
Gambogenic acid	143B, HOS	P53/SLC7A11/GPX4	SLC7A11, p53	GSH depletion; p53 signaling pathway activation; ROS generation; inhibition of cell proliferation; inducing ferroptosis and apoptosis	[285]

Pro-oxidative cancer therapies are a class of treatments designed to exploit the increased levels of oxidative stress typically found in cancer cells compared to normal cells. These therapies work by further increasing the levels of ROS within cancer cells, ultimately leading to cell death [286]. Some of the pro-oxidative drugs have been investigated in preclinical studies and clinical trials covering various aspects of oxidative stress and lipid peroxidation in cancer cells, including osteosarcoma [reviewed in [287]. Pro-oxidative therapies can also enhance the effectiveness of other cancer treatments. For example, they can sensitize cancer cells to chemotherapy or radiation therapy, making these treatments more potent [288].

Many chemotherapeutic drugs exert their anticancer effects through the direct or indirect induction of lipid peroxidation, which plays a significant role in the cytotoxic effects of certain chemotherapeutic agents. Certain chemotherapy drugs directly generate ROS within cancer cells. These ROS can overcome the antioxidant defenses of cancer cells, leading to oxidative stress and subsequent cell death. For example, doxorubicin and cisplatin are chemotherapy drugs that promote ROS formation as part of their mechanisms of action, which can induce lipid peroxidation and the formation of lipid peroxidation products, such as MDA and 4-HNE [276,289]. Many other drugs, like Paclitaxel and Vinblastine, arsenic trioxide, 5-fluorouracil, and tyrosine kinase inhibitors, such as Vorinostat and Sorafenib, induce oxidative stress by targeting cellular processes that result in ROS production, leading to the accumulation of ROS in cancer cells (reviewed in [290]). Anticancer drugs may have intrinsic ROS-generating activity but combining them with additional ROS-inducing agents can further increase ROS levels. When ROS-inducing agents are used in combination with anticancer drugs, they can potentiate the cytotoxic effects of the drugs, leading to enhanced cell death.

Cancer cells frequently upregulate de novo fatty acid synthesis to fulfill their high demand for lipids, essential for membrane formation, energy storage, and signaling molecules. Fatty acid synthase (FASN) is a key enzyme involved in de novo fatty acid synthesis. The inhibitors of FASN, such as cerulenin, orlistat, TVB-2640, and C75, have been developed and tested in preclinical and clinical studies [291]. Some therapeutic agents, such as Soraphen A, are oriented towards the inhibition of ACC, an enzyme that catalyzes the carboxylation of acetyl-CoA to malonyl-CoA, a precursor for fatty acid synthesis [291]. Other compounds targeting SCD1, CPT1, DGAT, and other key enzymes involved in fatty acid metabolism also hold promise as a cancer treatment strategy [267].

The specific impact of these drugs on lipid peroxidation and ROS can vary depending on the context, including the type of cancer being treated and the specific characteristics of the cancer cells. While most of these treatments are not specifically targeted at lipid peroxidation products or treatments for OS, their induction of oxidative stress and potential to induce lipid peroxidation may contribute to their anticancer effects in OS therapy. However, further research is needed to elucidate the specific mechanisms underlying lipid peroxidation induced by these drugs in OS cells and their therapeutic implications.

## 6. Conclusions

The results of many years of research, together with the most recent findings, allow us to conclude that redox signaling in osteosarcoma, similar to the other types of cancer, plays a crucial regulatory role in supporting the survival of transformed cells and their growth. Therefore, further research, in particular focused on the roles of lipid peroxidation, could help not only better understanding but also better prevention and therapies for osteosarcoma.

## Data Availability

Not applicable.
